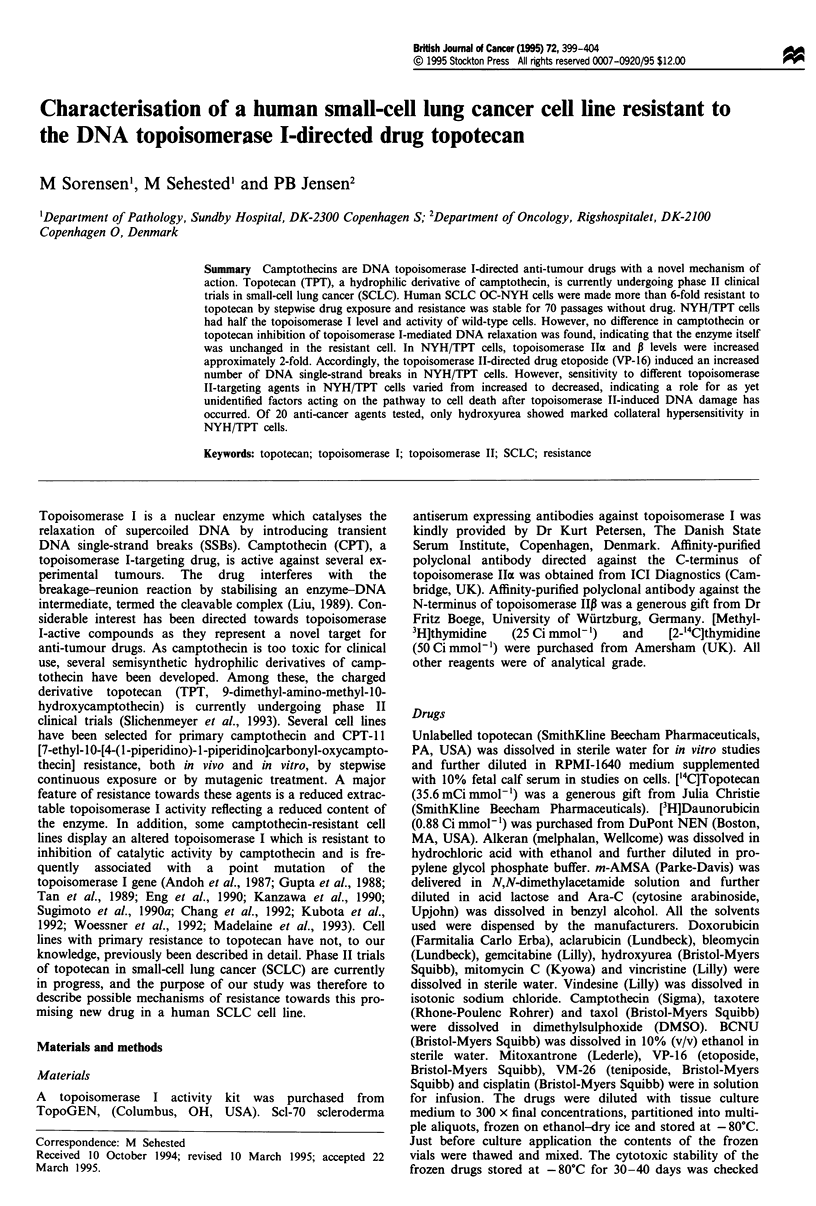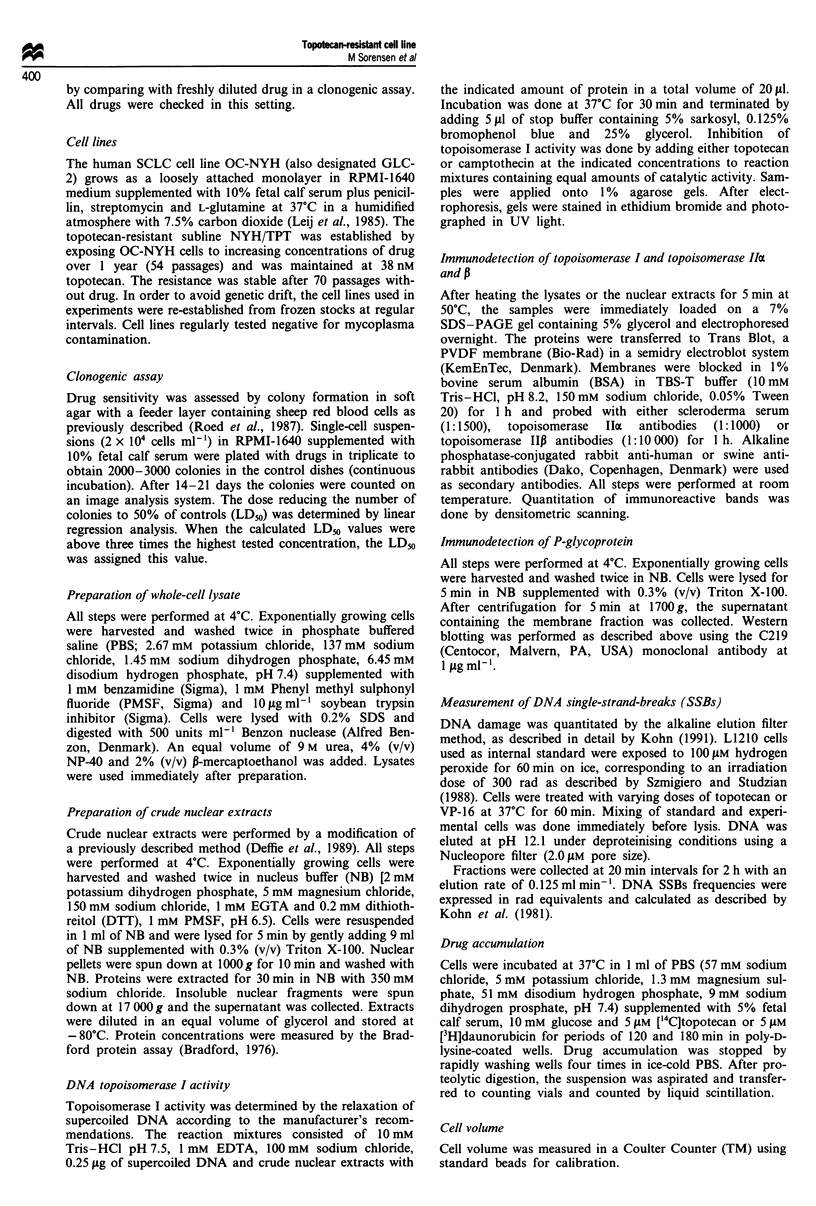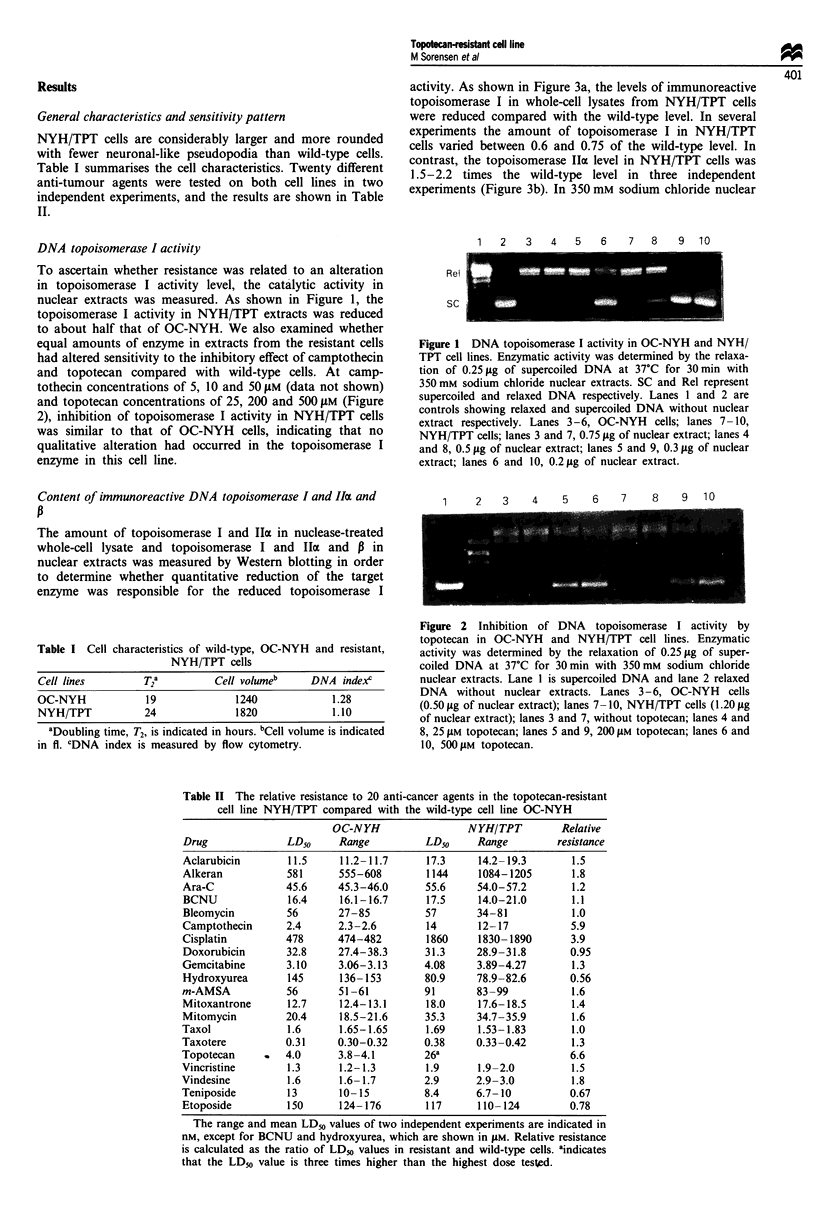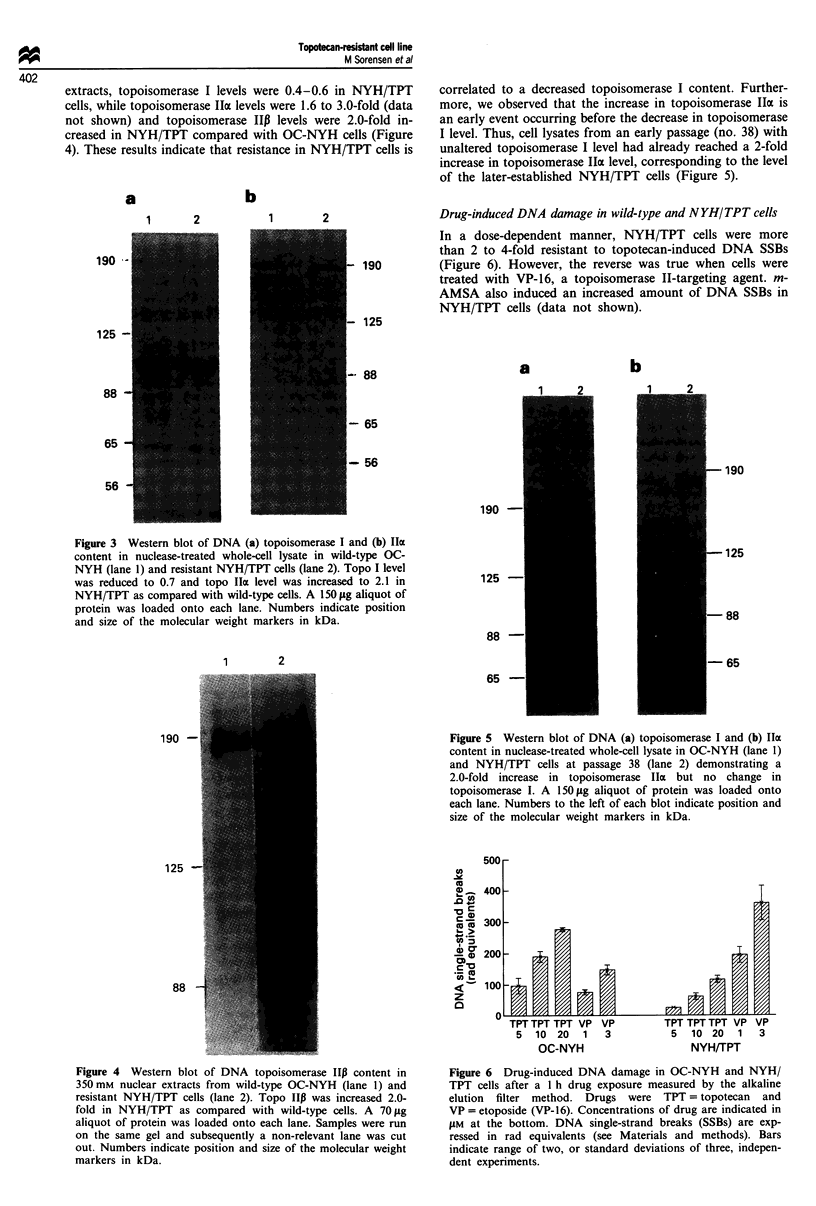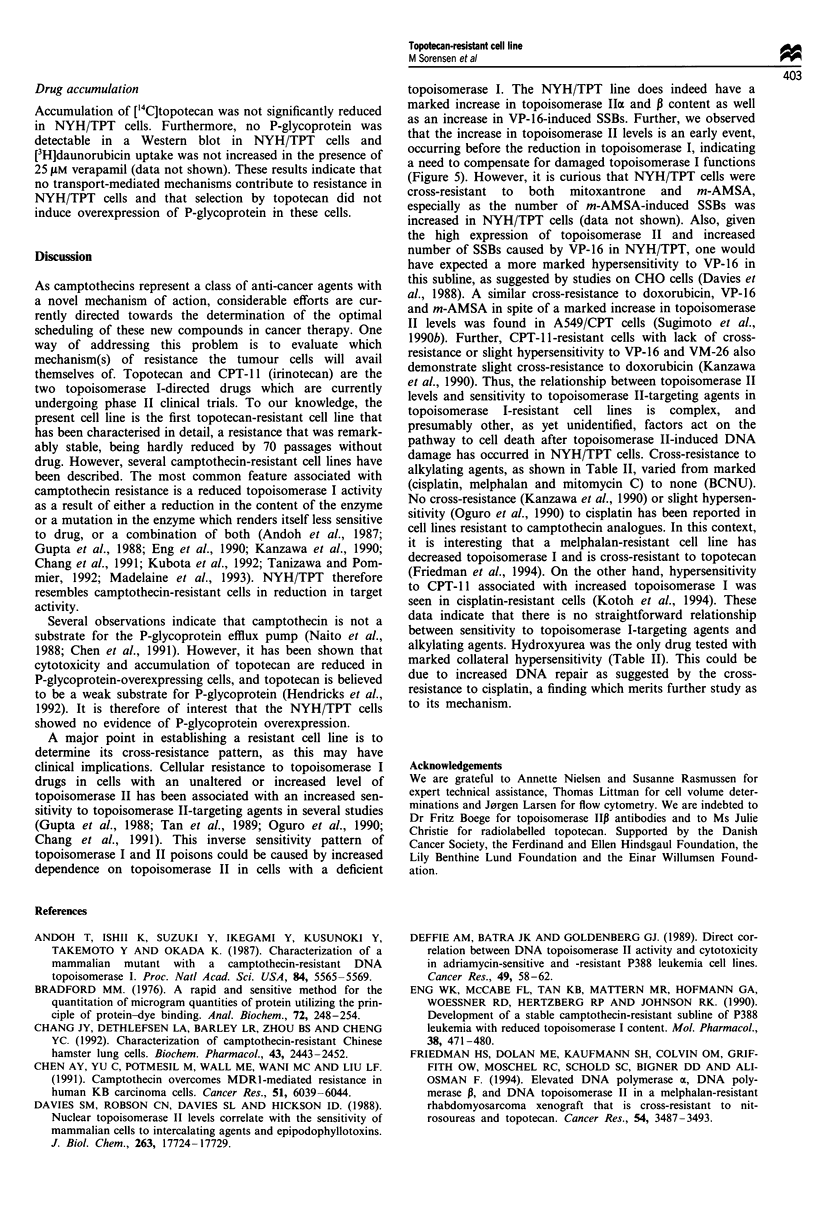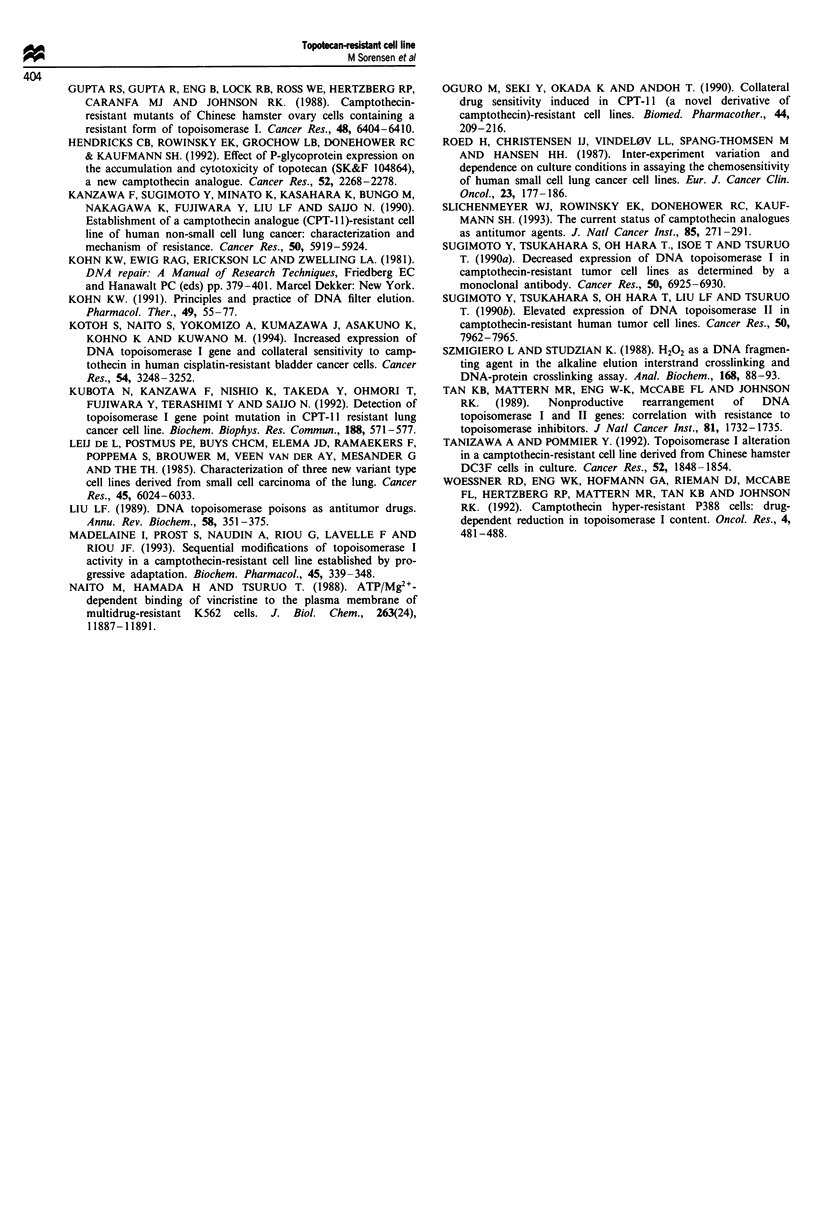# Characterisation of a human small-cell lung cancer cell line resistant to the DNA topoisomerase I-directed drug topotecan.

**DOI:** 10.1038/bjc.1995.345

**Published:** 1995-08

**Authors:** M. Sorensen, M. Sehested, P. B. Jensen

**Affiliations:** Department of Pathology, Sundby Hospital, Copenhagen, Denmark.

## Abstract

**Images:**


British Journal of Cancer (1995) 72, 399-404
© 1995 Stockton Press All rights reserved 0007-0920/95 $12.00

### Characterisation of a human small-cell lung cancer cell line resistant to the DNA topoisomerase I-directed drug topotecan

M Sorensen[1], M Sehested[1] and PB Jensen[2]

[1]*Department of Pathology, Sundby Hospital, DK-2300 Copenhagen S;* [2]*Department of Oncology, Rigshospitalet, DK-2100 Copenhagen O, Denmark*

**Summary** Camptothecins are DNA topoisomerase I-directed anti-tumour drugs with a novel mechanism of action. Topotecan (TPT), a hydrophilic derivative of camptothecin, is currently undergoing phase II clinical trials in small-cell lung cancer (SCLC). Human SCLC OC-NYH cells were made more than 6-fold resistant to topotecan by stepwise drug exposure and resistance was stable for 70 passages without drug. NYH/TPT cells had half the topoisomerase I level and activity of wild-type cells. However, no difference in camptothecin or topotecan inhibition of topoisomerase I-mediated DNA relaxation was found, indicating that the enzyme itself was unchanged in the resistant cell. In NYH/TPT cells, topoisomerase IIα and β levels were increased approximately 2-fold. Accordingly, the topoisomerase II-directed drug etoposide (VP-16) induced an increased number of DNA single-strand breaks in NYH/TPT cells. However, sensitivity to different topoisomerase II-targeting agents in NYH/TPT cells varied from increased to decreased, indicating a role for as yet unidentified factors acting on the pathway to cell death after topoisomerase II-induced DNA damage has occurred. Of 20 anti-cancer agents tested, only hydroxyurea showed marked collateral hypersensitivity in NYH/TPT cells.

**Keywords:** topotecan; topoisomerase I; topoisomerase II; SCLC; resistance

Topoisomerase I is a nuclear enzyme which catalyses the relaxation of supercoiled DNA by introducing transient DNA single-strand breaks (SSBs). Camptothecin (CPT), a topoisomerase I-targeting drug, is active against several experimental tumours. The drug interferes with the breakage–reunion reaction by stabilising an enzyme–DNA intermediate, termed the cleavable complex (Liu, 1989). Considerable interest has been directed towards topoisomerase I-active compounds as they represent a novel target for anti-tumour drugs. As camptothecin is too toxic for clinical use, several semisynthetic hydrophilic derivatives of camptothecin have been developed. Among these, the charged derivative topotecan (TPT, 9-dimethyl-amino-methyl-10-hydroxycamptothecin) is currently undergoing phase II clinical trials (Slichenmeyer *et al.*, 1993). Several cell lines have been selected for primary camptothecin and CPT-11 [7-ethyl-10-[4-(1-piperidino)-1-piperidino]carbonyl-oxycamptothecin] resistance, both *in vivo* and *in vitro*, by stepwise continuous exposure or by mutagenic treatment. A major feature of resistance towards these agents is a reduced extractable topoisomerase I activity reflecting a reduced content of the enzyme. In addition, some camptothecin-resistant cell lines display an altered topoisomerase I which is resistant to inhibition of catalytic activity by camptothecin and is frequently associated with a point mutation of the topoisomerase I gene (Andoh *et al.*, 1987; Gupta *et al.*, 1988; Tan *et al.*, 1989; Eng *et al.*, 1990; Kanzawa *et al.*, 1990; Sugimoto *et al.*, 1990*a*; Chang *et al.*, 1992; Kubota *et al.*, 1992; Woessner *et al.*, 1992; Madelaine *et al.*, 1993). Cell lines with primary resistance to topotecan have not, to our knowledge, previously been described in detail. Phase II trials of topotecan in small-cell lung cancer (SCLC) are currently in progress, and the purpose of our study was therefore to describe possible mechanisms of resistance towards this promising new drug in a human SCLC cell line.

### Materials and methods

### *Materials*

A topoisomerase I activity kit was purchased from TopoGEN, (Columbus, OH, USA). Scl-70 scleroderma antiserum expressing antibodies against topoisomerase I was kindly provided by Dr Kurt Petersen, The Danish State Serum Institute, Copenhagen, Denmark. Affinity-purified polyclonal antibody directed against the C-terminus of topoisomerase IIα was obtained from ICI Diagnostics (Cambridge, UK). Affinity-purified polyclonal antibody against the N-terminus of topoisomerase IIβ was a generous gift from Dr Fritz Boege, University of Würtzburg, Germany. [Methyl-$^3$H]thymidine (25 Ci mmol$^{-1}$) and [2-$^{14}$C]thymidine (50 Ci mmol$^{-1}$) were purchased from Amersham (UK). All other reagents were of analytical grade.

### *Drugs*

Unlabelled topotecan (SmithKline Beecham Pharmaceuticals, PA, USA) was dissolved in sterile water for *in vitro* studies and further diluted in RPMI-1640 medium supplemented with 10% fetal calf serum in studies on cells. [$^{14}$C]Topotecan (35.6 mCi mmol$^{-1}$) was a generous gift from Julia Christie (SmithKline Beecham Pharmaceuticals). [$^3$H]Daunorubicin (0.88 Ci mmol$^{-1}$) was purchased from DuPont NEN (Boston, MA, USA). Alkeran (melphalan, Wellcome) was dissolved in hydrochloric acid with ethanol and further diluted in propylene glycol phosphate buffer. *m*-AMSA (Parke-Davis) was delivered in *N,N*-dimethylacetamide solution and further diluted in acid lactose and Ara-C (cytosine arabinoside, Upjohn) was dissolved in benzyl alcohol. All the solvents used were dispensed by the manufacturers. Doxorubicin (Farmitalia Carlo Erba), aclarubicin (Lundbeck), bleomycin (Lundbeck), gemcitabine (Lilly), hydroxyurea (Bristol-Myers Squibb), mitomycin C (Kyowa) and vincristine (Lilly) were dissolved in sterile water. Vindesine (Lilly) was dissolved in isotonic sodium chloride. Camptothecin (Sigma), taxotere (Rhone-Poulenc Rohrer) and taxol (Bristol-Myers Squibb) were dissolved in dimethylsulphoxide (DMSO). BCNU (Bristol-Myers Squibb) was dissolved in 10% (v/v) ethanol in sterile water. Mitoxantrone (Lederle), VP-16 (etoposide, Bristol-Myers Squibb), VM-26 (teniposide, Bristol-Myers Squibb) and cisplatin (Bristol-Myers Squibb) were in solution for infusion. The drugs were diluted with tissue culture medium to 300 × final concentrations, partitioned into multiple aliquots, frozen on ethanol–dry ice and stored at −80°C. Just before culture application the contents of the frozen vials were thawed and mixed. The cytotoxic stability of the frozen drugs stored at −80°C for 30–40 days was checked

Correspondence: M Sehested
Received 10 October 1994; revised 10 March 1995; accepted 22 March 1995.

by comparing with freshly diluted drug in a clonogenic assay. All drugs were checked in this setting.

*Cell lines*

The human SCLC cell line OC-NYH (also designated GLC-2) grows as a loosely attached monolayer in RPMI-1640 medium supplemented with 10% fetal calf serum plus penicillin, streptomycin and L-glutamine at 37°C in a humidified atmosphere with 7.5% carbon dioxide (Leij *et al.*, 1985). The topotecan-resistant subline NYH/TPT was established by exposing OC-NYH cells to increasing concentrations of drug over 1 year (54 passages) and was maintained at 38 nM topotecan. The resistance was stable after 70 passages without drug. In order to avoid genetic drift, the cell lines used in experiments were re-established from frozen stocks at regular intervals. Cell lines regularly tested negative for mycoplasma contamination.

*Clonogenic assay*

Drug sensitivity was assessed by colony formation in soft agar with a feeder layer containing sheep red blood cells as previously described (Roed *et al.*, 1987). Single-cell suspensions ($2 \times 10^4$ cells $ml^{-1}$) in RPMI-1640 supplemented with 10% fetal calf serum were plated with drugs in triplicate to obtain 2000–3000 colonies in the control dishes (continuous incubation). After 14–21 days the colonies were counted on an image analysis system. The dose reducing the number of colonies to 50% of controls ($LD_{50}$) was determined by linear regression analysis. When the calculated $LD_{50}$ values were above three times the highest tested concentration, the $LD_{50}$ was assigned this value.

*Preparation of whole-cell lysate*

All steps were performed at 4°C. Exponentially growing cells were harvested and washed twice in phosphate buffered saline (PBS; 2.67 mM potassium chloride, 137 mM sodium chloride, 1.45 mM sodium dihydrogen phosphate, 6.45 mM disodium hydrogen phosphate, pH 7.4) supplemented with 1 mM benzamidine (Sigma), 1 mM Phenyl methyl sulphonyl fluoride (PMSF, Sigma) and 10 µg $ml^{-1}$ soybean trypsin inhibitor (Sigma). Cells were lysed with 0.2% SDS and digested with 500 units $ml^{-1}$ Benzon nuclease (Alfred Benzon, Denmark). An equal volume of 9 M urea, 4% (v/v) NP-40 and 2% (v/v) β-mercaptoethanol was added. Lysates were used immediately after preparation.

*Preparation of crude nuclear extracts*

Crude nuclear extracts were performed by a modification of a previously described method (Deffie *et al.*, 1989). All steps were performed at 4°C. Exponentially growing cells were harvested and washed twice in nucleus buffer (NB) [2 mM potassium dihydrogen phosphate, 5 mM magnesium chloride, 150 mM sodium chloride, 1 mM EGTA and 0.2 mM dithiothreitol (DTT), 1 mM PMSF, pH 6.5). Cells were resuspended in 1 ml of NB and were lysed for 5 min by gently adding 9 ml of NB supplemented with 0.3% (v/v) Triton X-100. Nuclear pellets were spun down at 1000 *g* for 10 min and washed with NB. Proteins were extracted for 30 min in NB with 350 mM sodium chloride. Insoluble nuclear fragments were spun down at 17 000 *g* and the supernatant was collected. Extracts were diluted in an equal volume of glycerol and stored at −80°C. Protein concentrations were measured by the Bradford protein assay (Bradford, 1976).

*DNA topoisomerase I activity*

Topoisomerase I activity was determined by the relaxation of supercoiled DNA according to the manufacturer's recommendations. The reaction mixtures consisted of 10 mM Tris–HCl pH 7.5, 1 mM EDTA, 100 mM sodium chloride, 0.25 µg of supercoiled DNA and crude nuclear extracts with the indicated amount of protein in a total volume of 20 µl. Incubation was done at 37°C for 30 min and terminated by adding 5 µl of stop buffer containing 5% sarkosyl, 0.125% bromophenol blue and 25% glycerol. Inhibition of topoisomerase I activity was done by adding either topotecan or camptothecin at the indicated concentrations to reaction mixtures containing equal amounts of catalytic activity. Samples were applied onto 1% agarose gels. After electrophoresis, gels were stained in ethidium bromide and photographed in UV light.

*Immunodetection of topoisomerase I and topoisomerase IIα and β*

After heating the lysates or the nuclear extracts for 5 min at 50°C, the samples were immediately loaded on a 7% SDS–PAGE gel containing 5% glycerol and electrophoresed overnight. The proteins were transferred to Trans Blot, a PVDF membrane (Bio-Rad) in a semidry electroblot system (KemEnTec, Denmark). Membranes were blocked in 1% bovine serum albumin (BSA) in TBS-T buffer (10 mM Tris–HCl, pH 8.2, 150 mM sodium chloride, 0.05% Tween 20) for 1 h and probed with either scleroderma serum (1:1500), topoisomerase IIα antibodies (1:1000) or topoisomerase IIβ antibodies (1:10 000) for 1 h. Alkaline phosphatase-conjugated rabbit anti-human or swine anti-rabbit antibodies (Dako, Copenhagen, Denmark) were used as secondary antibodies. All steps were performed at room temperature. Quantitation of immunoreactive bands was done by densitometric scanning.

*Immunodetection of P-glycoprotein*

All steps were performed at 4°C. Exponentially growing cells were harvested and washed twice in NB. Cells were lysed for 5 min in NB supplemented with 0.3% (v/v) Triton X-100. After centrifugation for 5 min at 1700 *g*, the supernatant containing the membrane fraction was collected. Western blotting was performed as described above using the C219 (Centocor, Malvern, PA, USA) monoclonal antibody at 1 µg $ml^{-1}$.

*Measurement of DNA single-strand-breaks (SSBs)*

DNA damage was quantitated by the alkaline elution filter method, as described in detail by Kohn (1991). L1210 cells used as internal standard were exposed to 100 µM hydrogen peroxide for 60 min on ice, corresponding to an irradiation dose of 300 rad as described by Szmigiero and Studzian (1988). Cells were treated with varying doses of topotecan or VP-16 at 37°C for 60 min. Mixing of standard and experimental cells was done immediately before lysis. DNA was eluted at pH 12.1 under deproteinising conditions using a Nucleopore filter (2.0 µM pore size).

Fractions were collected at 20 min intervals for 2 h with an elution rate of 0.125 ml $min^{-1}$. DNA SSBs frequencies were expressed in rad equivalents and calculated as described by Kohn *et al.* (1981).

*Drug accumulation*

Cells were incubated at 37°C in 1 ml of PBS (57 mM sodium chloride, 5 mM potassium chloride, 1.3 mM magnesium sulphate, 51 mM disodium hydrogen phosphate, 9 mM sodium dihydrogen prosphate, pH 7.4) supplemented with 5% fetal calf serum, 10 mM glucose and 5 µM [$^{14}C$]topotecan or 5 µM [$^{3}H$]daunorubicin for periods of 120 and 180 min in poly-D-lysine-coated wells. Drug accumulation was stopped by rapidly washing wells four times in ice-cold PBS. After proteolytic digestion, the suspension was aspirated and transferred to counting vials and counted by liquid scintillation.

*Cell volume*

Cell volume was measured in a Coulter Counter (TM) using standard beads for calibration.

### Results

### *General characteristics and sensitivity pattern*

NYH/TPT cells are considerably larger and more rounded with fewer neuronal-like pseudopodia than wild-type cells. Table I summarises the cell characteristics. Twenty different anti-tumour agents were tested on both cell lines in two independent experiments, and the results are shown in Table II.

### *DNA topoisomerase I activity*

To ascertain whether resistance was related to an alteration in topoisomerase I activity level, the catalytic activity in nuclear extracts was measured. As shown in Figure 1, the topoisomerase I activity in NYH/TPT extracts was reduced to about half that of OC-NYH. We also examined whether equal amounts of enzyme in extracts from the resistant cells had altered sensitivity to the inhibitory effect of camptothecin and topotecan compared with wild-type cells. At camptothecin concentrations of 5, 10 and 50 μM (data not shown) and topotecan concentrations of 25, 200 and 500 μM (Figure 2), inhibition of topoisomerase I activity in NYH/TPT cells was similar to that of OC-NYH cells, indicating that no qualitative alteration had occurred in the topoisomerase I enzyme in this cell line.

### *Content of immunoreactive DNA topoisomerase I and IIα and β*

The amount of topoisomerase I and IIα in nuclease-treated whole-cell lysate and topoisomerase I and IIα and β in nuclear extracts was measured by Western blotting in order to determine whether quantitative reduction of the target enzyme was responsible for the reduced topoisomerase I activity. As shown in Figure 3a, the levels of immunoreactive topoisomerase I in whole-cell lysates from NYH/TPT cells were reduced compared with the wild-type level. In several experiments the amount of topoisomerase I in NYH/TPT cells varied between 0.6 and 0.75 of the wild-type level. In contrast, the topoisomerase IIα level in NYH/TPT cells was 1.5–2.2 times the wild-type level in three independent experiments (Figure 3b). In 350 mM sodium chloride nuclear

**Table I** Cell characteristics of wild-type, OC-NYH and resistant, NYH/TPT cells

| *Cell lines* | $T_2$[a] | *Cell volume*[b] | *DNA index*[c] |
|---|---|---|---|
| OC-NYH | 19 | 1240 | 1.28 |
| NYH/TPT | 24 | 1820 | 1.10 |

[a]Doubling time, $T_2$, is indicated in hours. [b]Cell volume is indicated in fl. [c]DNA index is measured by flow cytometry.

**Figure 1** DNA topoisomerase I activity in OC-NYH and NYH/TPT cell lines. Enzymatic activity was determined by the relaxation of 0.25 μg of supercoiled DNA at 37°C for 30 min with 350 mM sodium chloride nuclear extracts. SC and Rel represent supercoiled and relaxed DNA respectively. Lanes 1 and 2 are controls showing relaxed and supercoiled DNA without nuclear extract respectively. Lanes 3–6, OC-NYH cells; lanes 7–10, NYH/TPT cells; lanes 3 and 7, 0.75 μg of nuclear extract; lanes 4 and 8, 0.5 μg of nuclear extract; lanes 5 and 9, 0.3 μg of nuclear extract; lanes 6 and 10, 0.2 μg of nuclear extract.

**Figure 2** Inhibition of DNA topoisomerase I activity by topotecan in OC-NYH and NYH/TPT cell lines. Enzymatic activity was determined by the relaxation of 0.25 μg of supercoiled DNA at 37°C for 30 min with 350 mM sodium chloride nuclear extracts. Lane 1 is supercoiled DNA and lane 2 relaxed DNA without nuclear extracts. Lanes 3–6, OC-NYH cells (0.50 μg of nuclear extract); lanes 7–10, NYH/TPT cells (1.20 μg of nuclear extract); lanes 3 and 7, without topotecan; lanes 4 and 8, 25 μM topotecan; lanes 5 and 9, 200 μM topotecan; lanes 6 and 10, 500 μM topotecan.

**Table II** The relative resistance to 20 anti-cancer agents in the topotecan-resistant cell line NYH/TPT compared with the wild-type cell line OC-NYH

| | *OC-NYH* | | *NYH/TPT* | | *Relative* |
|---|---|---|---|---|---|
| *Drug* | $LD_{50}$ | *Range* | $LD_{50}$ | *Range* | *resistance* |
| Aclarubicin | 11.5 | 11.2–11.7 | 17.3 | 14.2–19.3 | 1.5 |
| Alkeran | 581 | 555–608 | 1144 | 1084–1205 | 1.8 |
| Ara-C | 45.6 | 45.3–46.0 | 55.6 | 54.0–57.2 | 1.2 |
| BCNU | 16.4 | 16.1–16.7 | 17.5 | 14.0–21.0 | 1.1 |
| Bleomycin | 56 | 27–85 | 57 | 34–81 | 1.0 |
| Camptothecin | 2.4 | 2.3–2.6 | 14 | 12–17 | 5.9 |
| Cisplatin | 478 | 474–482 | 1860 | 1830–1890 | 3.9 |
| Doxorubicin | 32.8 | 27.4–38.3 | 31.3 | 28.9–31.8 | 0.95 |
| Gemcitabine | 3.10 | 3.06–3.13 | 4.08 | 3.89–4.27 | 1.3 |
| Hydroxyurea | 145 | 136–153 | 80.9 | 78.9–82.6 | 0.56 |
| *m*-AMSA | 56 | 51–61 | 91 | 83–99 | 1.6 |
| Mitoxantrone | 12.7 | 12.4–13.1 | 18.0 | 17.6–18.5 | 1.4 |
| Mitomycin | 20.4 | 18.5–21.6 | 35.3 | 34.7–35.9 | 1.6 |
| Taxol | 1.6 | 1.65–1.65 | 1.69 | 1.53–1.83 | 1.0 |
| Taxotere | 0.31 | 0.30–0.32 | 0.38 | 0.33–0.42 | 1.3 |
| Topotecan | 4.0 | 3.8–4.1 | 26[a] | | 6.6 |
| Vincristine | 1.3 | 1.2–1.3 | 1.9 | 1.9–2.0 | 1.5 |
| Vindesine | 1.6 | 1.6–1.7 | 2.9 | 2.9–3.0 | 1.8 |
| Teniposide | 13 | 10–15 | 8.4 | 6.7–10 | 0.67 |
| Etoposide | 150 | 124–176 | 117 | 110–124 | 0.78 |

The range and mean $LD_{50}$ values of two independent experiments are indicated in nM, except for BCNU and hydroxyurea, which are shown in μM. Relative resistance is calculated as the ratio of $LD_{50}$ values in resistant and wild-type cells. [a]indicates that the $LD_{50}$ value is three times higher than the highest dose tested.

extracts, topoisomerase I levels were 0.4–0.6 in NYH/TPT cells, while topoisomerase IIα levels were 1.6 to 3.0-fold (data not shown) and topoisomerase IIβ levels were 2.0-fold increased in NYH/TPT compared with OC-NYH cells (Figure 4). These results indicate that resistance in NYH/TPT cells is correlated to a decreased topoisomerase I content. Furthermore, we observed that the increase in topoisomerase IIα is an early event occurring before the decrease in topoisomerase I level. Thus, cell lysates from an early passage (no. 38) with unaltered topoisomerase I level had already reached a 2-fold increase in topoisomerase IIα level, corresponding to the level of the later-established NYH/TPT cells (Figure 5).

**Figure 3** Western blot of DNA (**a**) topoisomerase I and (**b**) IIα content in nuclease-treated whole-cell lysate in wild-type OC-NYH (lane 1) and resistant NYH/TPT cells (lane 2). Topo I level was reduced to 0.7 and topo IIα level was increased to 2.1 in NYH/TPT as compared with wild-type cells. A 150 μg aliquot of protein was loaded onto each lane. Numbers indicate position and size of the molecular weight markers in kDa.

**Figure 4** Western blot of DNA topoisomerase IIβ content in 350 mM nuclear extracts from wild-type OC-NYH (lane 1) and resistant NYH/TPT cells (lane 2). Topo IIβ was increased 2.0-fold in NYH/TPT as compared with wild-type cells. A 70 μg aliquot of protein was loaded onto each lane. Samples were run on the same gel and subsequently a non-relevant lane was cut out. Numbers indicate position and size of the molecular weight markers in kDa.

### *Drug-induced DNA damage in wild-type and NYH/TPT cells*

In a dose-dependent manner, NYH/TPT cells were more than 2 to 4-fold resistant to topotecan-induced DNA SSBs (Figure 6). However, the reverse was true when cells were treated with VP-16, a topoisomerase II-targeting agent. *m*-AMSA also induced an increased amount of DNA SSBs in NYH/TPT cells (data not shown).

**Figure 5** Western blot of DNA (**a**) topoisomerase I and (**b**) IIα content in nuclease-treated whole-cell lysate in OC-NYH (lane 1) and NYH/TPT cells at passage 38 (lane 2) demonstrating a 2.0-fold increase in topoisomerase IIα but no change in topoisomerase I. A 150 μg aliquot of protein was loaded onto each lane. Numbers to the left of each blot indicate position and size of the molecular weight markers in kDa.

**Figure 6** Drug-induced DNA damage in OC-NYH and NYH/TPT cells after a 1 h drug exposure measured by the alkaline elution filter method. Drugs were TPT = topotecan and VP = etoposide (VP-16). Concentrations of drug are indicated in μM at the bottom. DNA single-strand breaks (SSBs) are expressed in rad equivalents (see Materials and methods). Bars indicate range of two, or standard deviations of three, independent experiments.

*Drug accumulation*

Accumulation of [$^{14}$C]topotecan was not significantly reduced in NYH/TPT cells. Furthermore, no P-glycoprotein was detectable in a Western blot in NYH/TPT cells and [$^{3}$H]daunorubicin uptake was not increased in the presence of 25 $\mu$M verapamil (data not shown). These results indicate that no transport-mediated mechanisms contribute to resistance in NYH/TPT cells and that selection by topotecan did not induce overexpression of P-glycoprotein in these cells.

### Discussion

As camptothecins represent a class of anti-cancer agents with a novel mechanism of action, considerable efforts are currently directed towards the determination of the optimal scheduling of these new compounds in cancer therapy. One way of addressing this problem is to evaluate which mechanism(s) of resistance the tumour cells will avail themselves of. Topotecan and CPT-11 (irinotecan) are the two topoisomerase I-directed drugs which are currently undergoing phase II clinical trials. To our knowledge, the present cell line is the first topotecan-resistant cell line that has been characterised in detail, a resistance that was remarkably stable, being hardly reduced by 70 passages without drug. However, several camptothecin-resistant cell lines have been described. The most common feature associated with camptothecin resistance is a reduced topoisomerase I activity as a result of either a reduction in the content of the enzyme or a mutation in the enzyme which renders itself less sensitive to drug, or a combination of both (Andoh *et al.*, 1987; Gupta *et al.*, 1988; Eng *et al.*, 1990; Kanzawa *et al.*, 1990; Chang *et al.*, 1991; Kubota *et al.*, 1992; Tanizawa and Pommier, 1992; Madelaine *et al.*, 1993). NYH/TPT therefore resembles camptothecin-resistant cells in reduction in target activity.

Several observations indicate that camptothecin is not a substrate for the P-glycoprotein efflux pump (Naito *et al.*, 1988; Chen *et al.*, 1991). However, it has been shown that cytotoxicity and accumulation of topotecan are reduced in P-glycoprotein-overexpressing cells, and topotecan is believed to be a weak substrate for P-glycoprotein (Hendricks *et al.*, 1992). It is therefore of interest that the NYH/TPT cells showed no evidence of P-glycoprotein overexpression.

A major point in establishing a resistant cell line is to determine its cross-resistance pattern, as this may have clinical implications. Cellular resistance to topoisomerase I drugs in cells with an unaltered or increased level of topoisomerase II has been associated with an increased sensitivity to topoisomerase II-targeting agents in several studies (Gupta *et al.*, 1988; Tan *et al.*, 1989; Oguro *et al.*, 1990; Chang *et al.*, 1991). This inverse sensitivity pattern of topoisomerase I and II poisons could be caused by increased dependence on topoisomerase II in cells with a deficient topoisomerase I. The NYH/TPT line does indeed have a marked increase in topoisomerase II$\alpha$ and $\beta$ content as well as an increase in VP-16-induced SSBs. Further, we observed that the increase in topoisomerase II levels is an early event, occurring before the reduction in topoisomerase I, indicating a need to compensate for damaged topoisomerase I functions (Figure 5). However, it is curious that NYH/TPT cells were cross-resistant to both mitoxantrone and *m*-AMSA, especially as the number of *m*-AMSA-induced SSBs was increased in NYH/TPT cells (data not shown). Also, given the high expression of topoisomerase II and increased number of SSBs caused by VP-16 in NYH/TPT, one would have expected a more marked hypersensitivity to VP-16 in this subline, as suggested by studies on CHO cells (Davies *et al.*, 1988). A similar cross-resistance to doxorubicin, VP-16 and *m*-AMSA in spite of a marked increase in topoisomerase II levels was found in A549/CPT cells (Sugimoto *et al.*, 1990*b*). Further, CPT-11-resistant cells with lack of cross-resistance or slight hypersensitivity to VP-16 and VM-26 also demonstrate slight cross-resistance to doxorubicin (Kanzawa *et al.*, 1990). Thus, the relationship between topoisomerase II levels and sensitivity to topoisomerase II-targeting agents in topoisomerase I-resistant cell lines is complex, and presumably other, as yet unidentified, factors act on the pathway to cell death after topoisomerase II-induced DNA damage has occurred in NYH/TPT cells. Cross-resistance to alkylating agents, as shown in Table II, varied from marked (cisplatin, melphalan and mitomycin C) to none (BCNU). No cross-resistance (Kanzawa *et al.*, 1990) or slight hypersensitivity (Oguro *et al.*, 1990) to cisplatin has been reported in cell lines resistant to camptothecin analogues. In this context, it is interesting that a melphalan-resistant cell line has decreased topoisomerase I and is cross-resistant to topotecan (Friedman *et al.*, 1994). On the other hand, hypersensitivity to CPT-11 associated with increased topoisomerase I was seen in cisplatin-resistant cells (Kotoh *et al.*, 1994). These data indicate that there is no straightforward relationship between sensitivity to topoisomerase I-targeting agents and alkylating agents. Hydroxyurea was the only drug tested with marked collateral hypersensitivity (Table II). This could be due to increased DNA repair as suggested by the cross-resistance to cisplatin, a finding which merits further study as to its mechanism.

**Acknowledgements**
We are grateful to Annette Nielsen and Susanne Rasmussen for expert technical assistance, Thomas Littman for cell volume determinations and Jørgen Larsen for flow cytometry. We are indebted to Dr Fritz Boege for topoisomerase II$\beta$ antibodies and to Ms Julie Christie for radiolabelled topotecan. Supported by the Danish Cancer Society, the Ferdinand and Ellen Hindsgaul Foundation, the Lily Benthine Lund Foundation and the Einar Willumsen Foundation.

### References

ANDOH T, ISHII K, SUZUKI Y, IKEGAMI Y, KUSUNOKI Y, TAKEMOTO Y AND OKADA K. (1987). Characterization of a mammalian mutant with a camptothecin-resistant DNA topoisomerase I. *Proc. Natl Acad. Sci. USA*, **84**, 5565–5569.

BRADFORD MM. (1976). A rapid and sensitive method for the quantitation of microgram quantities of protein utilizing the principle of protein–dye binding. *Anal. Biochem.*, **72**, 248–254.

CHANG JY, DETHLEFSEN LA, BARLEY LR, ZHOU BS AND CHENG YC. (1992). Characterization of camptothecin-resistant Chinese hamster lung cells. *Biochem. Pharmacol.*, **43**, 2443–2452.

CHEN AY, YU C, POTMESIL M, WALL ME, WANI MC AND LIU LF. (1991). Camptothecin overcomes MDR1-mediated resistance in human KB carcinoma cells. *Cancer Res.*, **51**, 6039–6044.

DAVIES SM, ROBSON CN, DAVIES SL AND HICKSON ID. (1988). Nuclear topoisomerase II levels correlate with the sensitivity of mammalian cells to intercalating agents and epipodophyllotoxins. *J. Biol. Chem.*, **263**, 17724–17729.

DEFFIE AM, BATRA JK AND GOLDENBERG GJ. (1989). Direct correlation between DNA topoisomerase II activity and cytotoxicity in adriamycin-sensitive and -resistant P388 leukemia cell lines. *Cancer Res.*, **49**, 58–62.

ENG WK, MCCABE FL, TAN KB, MATTERN MR, HOFMANN GA, WOESSNER RD, HERTZBERG RP AND JOHNSON RK. (1990). Development of a stable camptothecin-resistant subline of P388 leukemia with reduced topoisomerase I content. *Mol. Pharmacol.*, **38**, 471–480.

FRIEDMAN HS, DOLAN ME, KAUFMANN SH, COLVIN OM, GRIFFITH OW, MOSCHEL RC, SCHOLD SC, BIGNER DD AND ALI-OSMAN F. (1994). Elevated DNA polymerase $\alpha$, DNA polymerase $\beta$, and DNA topoisomerase II in a melphalan-resistant rhabdomyosarcoma xenograft that is cross-resistant to nitrosoureas and topotecan. *Cancer Res.*, **54**, 3487–3493.

GUPTA RS, GUPTA R, ENG B, LOCK RB, ROSS WE, HERTZBERG RP, CARANFA MJ AND JOHNSON RK. (1988). Camptothecin-resistant mutants of Chinese hamster ovary cells containing a resistant form of topoisomerase I. *Cancer Res.*, **48**, 6404–6410.

HENDRICKS CB, ROWINSKY EK, GROCHOW LB, DONEHOWER RC & KAUFMANN SH. (1992). Effect of P-glycoprotein expression on the accumulation and cytotoxicity of topotecan (SK&F 104864), a new camptothecin analogue. *Cancer Res.*, **52**, 2268–2278.

KANZAWA F, SUGIMOTO Y, MINATO K, KASAHARA K, BUNGO M, NAKAGAWA K, FUJIWARA Y, LIU LF AND SAIJO N. (1990). Establishment of a camptothecin analogue (CPT-11)-resistant cell line of human non-small cell lung cancer: characterization and mechanism of resistance. *Cancer Res.*, **50**, 5919–5924.

KOHN KW, EWIG RAG, ERICKSON LC AND ZWELLING LA. (1981). *DNA repair: A Manual of Research Techniques*, Friedberg EC and Hanawalt PC (eds) pp. 379–401. Marcel Dekker: New York.

KOHN KW. (1991). Principles and practice of DNA filter elution. *Pharmacol. Ther.*, **49**, 55–77.

KOTOH S, NAITO S, YOKOMIZO A, KUMAZAWA J, ASAKUNO K, KOHNO K AND KUWANO M. (1994). Increased expression of DNA topoisomerase I gene and collateral sensitivity to camptothecin in human cisplatin-resistant bladder cancer cells. *Cancer Res.*, **54**, 3248–3252.

KUBOTA N, KANZAWA F, NISHIO K, TAKEDA Y, OHMORI T, FUJIWARA Y, TERASHIMI Y AND SAIJO N. (1992). Detection of topoisomerase I gene point mutation in CPT-11 resistant lung cancer cell line. *Biochem. Biophys. Res. Commun.*, **188**, 571–577.

LEIJ DE L, POSTMUS PE, BUYS CHCM, ELEMA JD, RAMAEKERS F, POPPEMA S, BROUWER M, VEEN VAN DER AY, MESANDER G AND THE TH. (1985). Characterization of three new variant type cell lines derived from small cell carcinoma of the lung. *Cancer Res.*, **45**, 6024–6033.

LIU LF. (1989). DNA topoisomerase poisons as antitumor drugs. *Annu. Rev. Biochem.*, **58**, 351–375.

MADELAINE I, PROST S, NAUDIN A, RIOU G, LAVELLE F AND RIOU JF. (1993). Sequential modifications of topoisomerase I activity in a camptothecin-resistant cell line established by progressive adaptation. *Biochem. Pharmacol.*, **45**, 339–348.

NAITO M, HAMADA H AND TSURUO T. (1988). ATP/$Mg^{2+}$-dependent binding of vincristine to the plasma membrane of multidrug-resistant K562 cells. *J. Biol. Chem.*, **263**(24), 11887–11891.

OGURO M, SEKI Y, OKADA K AND ANDOH T. (1990). Collateral drug sensitivity induced in CPT-11 (a novel derivative of camptothecin)-resistant cell lines. *Biomed. Pharmacother.*, **44**, 209–216.

ROED H, CHRISTENSEN IJ, VINDELØV LL, SPANG-THOMSEN M AND HANSEN HH. (1987). Inter-experiment variation and dependence on culture conditions in assaying the chemosensitivity of human small cell lung cancer cell lines. *Eur. J. Cancer Clin. Oncol.*, **23**, 177–186.

SLICHENMEYER WJ, ROWINSKY EK, DONEHOWER RC, KAUFMANN SH. (1993). The current status of camptothecin analogues as antitumor agents. *J. Natl Cancer Inst.*, **85**, 271–291.

SUGIMOTO Y, TSUKAHARA S, OH HARA T., ISOE T AND TSURUO T. (1990*a*). Decreased expression of DNA topoisomerase I in camptothecin-resistant tumor cell lines as determined by a monoclonal antibody. *Cancer Res.*, **50**, 6925–6930.

SUGIMOTO Y, TSUKAHARA S, OH HARA T, LIU LF AND TSURUO T. (1990*b*). Elevated expression of DNA topoisomerase II in camptothecin-resistant human tumor cell lines. *Cancer Res.*, **50**, 7962–7965.

SZMIGIERO L AND STUDZIAN K. (1988). $H_2O_2$ as a DNA fragmenting agent in the alkaline elution interstrand crosslinking and DNA-protein crosslinking assay. *Anal. Biochem.*, **168**, 88–93.

TAN KB, MATTERN MR, ENG W-K, MCCABE FL AND JOHNSON RK. (1989). Nonproductive rearrangement of DNA topoisomerase I and II genes: correlation with resistance to topoisomerase inhibitors. *J Natl Cancer Inst.*, **81**, 1732–1735.

TANIZAWA A AND POMMIER Y. (1992). Topoisomerase I alteration in a camptothecin-resistant cell line derived from Chinese hamster DC3F cells in culture. *Cancer Res.*, **52**, 1848–1854.

WOESSNER RD, ENG WK, HOFMANN GA, RIEMAN DJ, MCCABE FL, HERTZBERG RP, MATTERN MR, TAN KB AND JOHNSON RK. (1992). Camptothecin hyper-resistant P388 cells: drug-dependent reduction in topoisomerase I content. *Oncol. Res.*, **4**, 481–488.